# The therapeutic potential of targeting minimal residual disease in melanoma

**DOI:** 10.1002/ctm2.1197

**Published:** 2023-03-26

**Authors:** Riyaben P Patel, Pretashini M Somasundram, Lorey K. Smith, Karen E. Sheppard, Grant A. McArthur

**Affiliations:** ^1^ Cancer Research Division Peter MacCallum Cancer Centre Melbourne Victoria Australia; ^2^ Sir Peter MacCallum Department of Oncology University of Melbourne Parkville Victoria Australia; ^3^ Faculty of Medicine Dentistry and Health Sciences, University of Melbourne Parkville Victoria Australia; ^4^ Department of Medical Oncology Peter MacCallum Cancer Centre Melbourne Victoria Australia

**Keywords:** acquired resistance, BRAF/MEK inhibitors, combination treatment, cross‐resistance, immune checkpoint inhibitors, intrinsic resistance, melanoma, minimal residual disease

## Abstract

**Background:**

Cutaneous melanoma is a lethal form of skin cancer with morbidity and mortality rates highest amongst European, North American and Australasian populations. The developments of targeted therapies (TTs) directed at the oncogene *BRAF* and its downstream mediator MEK, and immune checkpoint inhibitors (ICI), have revolutionized the treatment of metastatic melanoma, improving patient outcomes. However, both TT and ICI have their limitations. Although TTs are associated with high initial response rates, these are typically short‐lived due to resistance. Conversely, although ICIs provide more durable responses, they have lower initial response rates. Due to these distinct yet complementary response profiles, it has been proposed that sequencing ICI with TT could lead to a high frequency of durable responses whilst circumventing the toxicity associated with combined ICI + TT treatment. However, several questions remain unanswered, including the mechanisms underpinning this synergy and the optimal sequencing strategy. The key to determining this is to uncover the biology of each phase of the therapeutic response.

**Aims and methods:**

In this review, we show that melanoma responds to TT and ICI in three phases: *early response*, *minimal residual disease (MRD)* and *disease progression*. We explore the effects of ICI and TT on melanoma cells and the tumour immune microenvironment, with a particular focus on MRD which is predicted to underpin the development of acquired resistance in the third phase of response.

**Conclusion:**

In doing so, we provide a new framework which may inform novel therapeutic approaches for melanoma, including optimal sequencing strategies and agents that target MRD, thereby ultimately improving clinical outcomes for patients.

## Graphical abstract

- We demonstrate that melanoma responds to targeted therapies (TTs) and immune checkpoint inhibitors (ICIs) in three phases: early response, minimal residual disease (MRD) and disease progression.
- The minimal residual disease of both TT and ICI is a nidus for the development of therapeutic resistance and, thus, warrants additional characterization of both tumour cells and their surrounding tumour immune microenvironment.
- Emerging new technologies, such as spatial transcriptomic, clonal evolution and high‐throughput single‐cell profiling, can be utilized to uncover novel therapeutic opportunities to target the minimal residual disease.

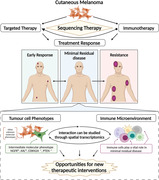

## 1 INTRODUCTION

Cutaneous melanoma is a highly aggressive cancer that presents a significant global burden of disease, with incidence rates highest in Australia and New Zealand (GLOBOCAN).^1^ Recent advances in characterizing the immunology and cancer biology of cutaneous melanoma have resulted in the development of immune and targeted therapies (TT). Although these therapeutic approaches have driven an improvement in the overall survival (OS) of patients, they are limited by mechanisms of both intrinsic and acquired resistances, which ultimately lead to disease progression. As such, there remains a pressing need to elucidate and overcome these mechanisms of resistance to improve patient outcomes.

### 1.1 Current treatment regimens

TTs include BRAF inhibitors (BRAFi) and MEK inhibitors (MEKi) which inhibit the commonly over‐activated mitogen‐activated protein kinase (MAPK) pathway, responsible for cell proliferation and survival in melanoma.^2^ These treatments produce high response rates that are typically short‐lived secondary to resistance.^3^, ^4^ Conversely, immune checkpoint inhibitors (ICI), which block cytotoxic T lymphocyte antigen‐4 (CTLA‐4) and Programmed cell death protein‐1/Programmed Death‐Ligand‐1 (PD‐1/PD‐L1), produce longer‐lived responses but have low response rates.^5^ Hence, the combination of TT + ICI has been proposed to increase the frequency of durable responses.^6^, ^7^ This has been confirmed by the IMspire150, Keynote‐22 and COMBI‐I trials, which have demonstrated improved progression‐free survival (PFS) with ICI + TT.^8^, ^9^, ^10^ Consequently, the FDA and other jurisdictions have approved triplet therapy (BRAFi + MEKi + ICI) as the first‐line treatment for *BRAF*‐mutant cutaneous melanoma. Notably, however, this improvement in efficacy comes at the price of higher toxicity rates (55%–58%) compared to TT (25%–33%) monotherapy.^8^, ^10^ To circumvent this, the approach of sequencing TT with ICI has been investigated (NCT02224781, NCT02631447). Ideally, this should be approached by understanding the biological effects of ICI and TT on tumour cells and the surrounding tumour immune microenvironment (TIME). In doing so, new therapeutic interventions may be developed that leverage this interaction to its maximum benefit. Herein, we aim to explore these therapeutic effects, with the aim of informing optimal treatment strategies.

## 2 TARGETED THERAPY

TTs have revolutionized the treatment landscape of advanced *BRAF* mutant melanoma. Genomic studies reveal that approximately 65%–90% of cutaneous melanoma harbour genetic events resulting in overactivation of the MAPK pathway.^1^, ^11^ Amongst the constituents of the MAPK pathway, BRAF is a critical player in the regulation of the cascade, and it is most prominently dysregulated in melanoma. *BRAF* somatic missense mutation where a valine amino acid is substituted for glutamic acid at exon 6 (*V600E*) is identified to occur in 50% of melanoma cases.^12^, ^13^ This mutation results in 700‐fold overactivation of the inherent BRAF kinase activity that enhances cell division and survival.^13^ In addition to *BRAF*, the second most common mutation in MAPK pathway is the small GTPase *NRAS* (25%), and the third is the tumour suppressor and the negative regulator of RAS, *neurofibromin 1* (NF1) (14%); both these mutations lead to the activation of the MAPK pathway and increased ERK signalling.^14^ Hence, targeting the most overactivated protein, BRAF and its downstream modulator MEK, TT mediates tumour regression in responders through MAPK blockade. In patients with *BRAF* mutant melanoma, the combination of BRAFi and MEKi has shown improvements in median OS from 6–9 to 22–33 months.^15^

**FIGURE 1:**
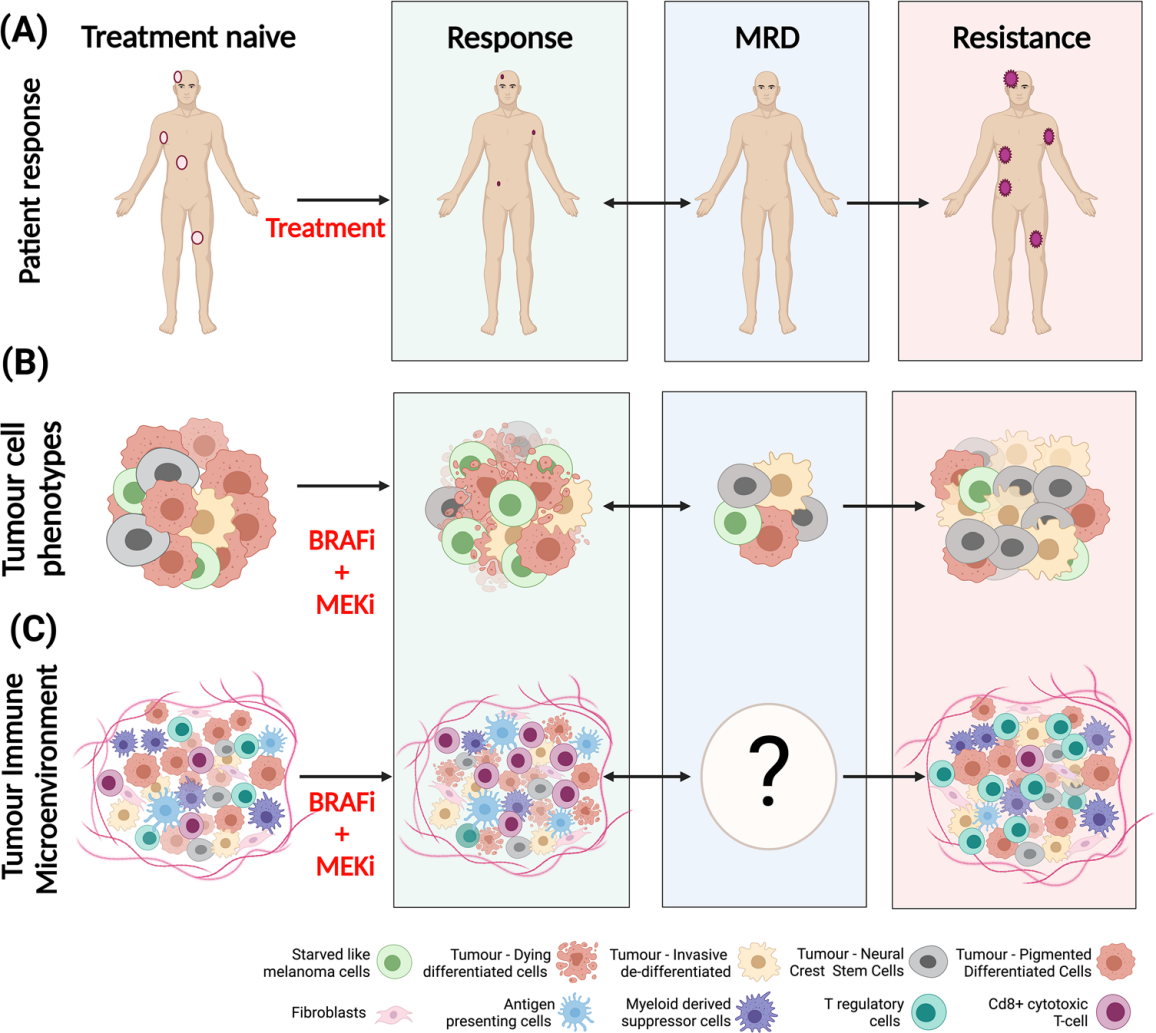
Response of cutaneous BRAF‐mutant melanoma to targeted therapy: (A) phases of the therapeutic response to targeted therapy. Cutaneous melanoma responds to targeted therapy in three phases. The first phase is defined by initial tumour regression upon exposure to therapy. The second phase is defined by minimal residual disease (MRD), whereby residual cancer cells persist whilst under therapeutic pressure, due to acquired genetic and non‐genetic changes. The third phase is defined by disease progression, whereby persistent cells of the MRD expand due to a growth and survival advantage; (B) non‐genetic mechanisms of acquired resistance. Melanoma cells undergo epigenetic and transcriptomic changes which result in the establishment of distinct melanoma phenotypes, thereby allowing cells to adapt to stressors within the tumour microenvironment. The treatment naïve phase is initially dominated by the pigmented differentiated cell phenotype. Upon therapeutic exposure to BRAFi + MEKi, non‐genetic changes result in the emergence of de‐differentiated cell phenotypes, including NCSCs and invasive cells, which ultimately drive resistance to targeted therapy. Genetic mechanisms of persistence may also exist; (C) changes within the tumour immune microenvironment (TIME) during each phase of the therapeutic response. The TIME plays a vital role in dictating therapeutic responses to targeted therapy. Treatment naïve melanoma is classically considered to be ‘immunologically hot’, dominated by immune stimulatory cell types, with marginal infiltrates of immune suppressive cells. Upon exposure to BRAFi + MEKi, this immunostimulatory TIME is further enriched and is characterized by increased infiltrates of cytotoxic Cd8+ T‐cells and increased antigen presentation through the upregulation of MHC I. Little is known about the composition of the TIME during the MRD phase. During the acquired resistance phase, the balance is tipped in favour of an immunosuppressive TIME characterized by increased T‐reg cells and reduced cytotoxic T‐cells and antigen‐presenting cells.

BRAF mutant melanoma is known to respond to TT in three phases (Figure 1A). The *first phase* is characterized by early response whereby ∼80% of patients undergo an initial phase of tumour regression. Conversely, ∼20% of patients fail to respond to TT and ultimately undergo tumour progression.^4^, ^16^ The *second phase* is characterized by minimal residual disease (MRD) whereby a portion of cancer cells undergo genetic and non‐genetic changes allowing them to survive therapeutic pressure in a state of tolerance/persistence.^17^, ^18^, ^19^ The *third phase* is characterized by drug resistance whereby MRD persister cells regain proliferative capacity and expand, ultimately driving disease progression. These changes are accompanied by a transformation in the TIME.^20^ Accordingly, the *second phase* is the nidus for the development of acquired resistance in the *third phase*. This highlights the importance of characterizing the genetic, non‐genetic and TIME changes during MRD to facilitate the creation of therapeutic strategies that circumvent the development of resistance.

### 2.1 First phase: responders versus non‐responders

The disease of responders is highly dependent on the MAPK pathway for its growth and survival. As such, through inhibition of BRAF and its downstream modulator MEK, TTs mediate tumour regression in responders through MAPK blockade.

Conversely, non‐responders harbour loss of tumour suppressor genes or activation of oncogenes which enables cell cycle entry through the upregulation of alternate signalling pathways such as phosphoinositide 3‐kinase (PI3K) or overactivation of MAPK pathways through alternate means.^20^, ^21^ In melanocytes, cyclin D1 regulates proliferation downstream of MAPK through activation of cyclin‐dependent kinase 4 (CDK4). *Cyclin D1* overexpression, especially when coupled with *CDK4* overexpression/activating mutations, has been found to confer intrinsic resistance to BRAFi.^22^ An additional innate mechanism conferring resistance involves activating mutations in the GTP binding protein *RAC1*, which mediates cell proliferation through MAPK^23^ and hepatocyte growth factor released by stromal cells in the tumour microenvironment which upregulates MAPK and PI3K pathways in melanoma cells.^24^ Therefore, having alternate activations besides the MAPK renders the non‐responders to be insensitive to MAPK blockade.

### 2.2 Second phase: minimal residual disease

The drug‐tolerant MRD phase is enabled by non‐genetic and genetic changes which confer a survival advantage.

Non‐genetic changes involve remodelling of tumour cells to adapt to stressors in their environment such as drug exposure. These changes result in the establishment of distinct melanoma phenotypes that have the capacity for adaptive switching between phenotypes (Figure 1B).^16^ Single‐cell RNA sequencing of malignant melanoma cells isolated from patient‐derived xenograft (PDX) models and human melanoma cell lines have identified at least four phenotypes: starved‐like melanoma cells (SMCs), neural crest stem cells (NCSCs) and invasive cells and differentiated cells.^16^ Experimental systems demonstrate that treatment‐naive melanoma is initially dominated by the differentiated phenotype which is defined by high expression levels of microphthalmia‐associated transcription factor (MITF).^25^ This transcription factor is a key regulator of genes involved in melanogenesis.^26^ During the first phase, after an initiation of therapy, there is a reduction in tumour growth with reduced numbers of differentiated cells. Furthermore, there is an emergence of SMCs due to the metabolic changes induced by early therapy exposure.^16^ With continued therapy, phenotypes emerge, including the NCSCs and invasive cells which persist under drug pressure.^16^ NCSCs have lower expression levels of *MITF* but higher expression levels of *AXL*, nerve growth factor receptor (*NGFR*) and SRY‐Box Transcription Factor 10 (*SOX10*). Both SOX10 and NGFR are key markers of neural crest lineage stem cells essential for melanocyte development.^16^, ^27^, ^28^ AXL is a receptor tyrosine kinase (RTK) characteristic of epithelial‐to‐mesenchymal transition. Conversely, the invasive phenotype is characterized by low expression levels of *MITF* and *SOX10*, though *AXL* levels remain high.^27^, ^28^, ^29^

Other persister cells within the MRD harbour genetic mutations resulting in the establishment of molecularly distinctive subclones.^25^ These mutations, which confer enhanced survival and proliferative capacity, allow the subclones to grow under drug pressure, thereby enabling therapeutic escape and acquired resistance.

### 2.3 Third phase: acquired resistance

The changes that occur during MRD ultimately enable the acquisition of resistance to TT and melanoma relapse. In particular, the NCSC and invasive phenotypes that emerge during MRD due to non‐genetic changes expand and subsequently drive disease progression in the third phase.^16^, ^28^ Interestingly, a study conducted in a PDX model of *BRAF* mutant melanoma demonstrated that NCSC depletion from MRD prevented the development of non‐genetic mechanisms of acquired resistance, leaving persistent cells dependent on de novo genetic mechanisms.^17^

Significant intra‐ and inter‐tumour heterogeneities have been found in the mechanisms of acquired genetic resistance.^30^ Reactivation of the MAPK pathway is present in 70% of patients who progress on TT,^20^ achieved through either upregulation or activating mutations of positive regulators of the MAPK pathway (*BRAF*, *MEK1, MEK2* and *NRAS*) or downregulation of negative regulators such as *NF1*,^31^, ^32^ ultimately driving melanoma proliferation and survival. Overactivation of the PI3K pathway due to a gain‐of‐function mutation in the positive regulator *AKT1* has also been found in approximately 20% of patients on progression.^33^ Additional mechanisms include alterations in cell‐cycle regulating proteins and RTKs.^34^

Hence, this heterogeneity highlights the challenge in developing a uniform therapeutic strategy for patients upon progression, thus emphasizing the importance of treatment before the acquisition of resistance.

### 2.4 Targeted therapy and the tumour immune microenvironment

In addition to the effects of TTs on the intrinsic biology of melanoma cells, preclinical and clinical studies also indicate that TTs influence the composition of the TIME at different phases of treatment response (Figure 1C).

#### 2.4.1 First phase

The early phase of TT response is characterized by an immune‐stimulatory TIME. TTs increase MHC class I expression and tumour antigen presentation^35^, ^36^ which promotes the expansion and migration of immune effector and cytotoxic cells into the tumour.^37^ This enhances the recognition of tumour antigens by cytotoxic T‐cells ultimately increasing overall antitumour activity.^38^, ^39^ Furthermore, pre‐clinical studies using mouse melanoma models have revealed that TT reduced infiltration of immunosuppressive cell types, including T‐regulatory cells (T‐reg) and myeloid‐derived suppressor cells,^40^ and reduced expression levels of immunosuppressive cytokines (e.g. IL‐10, VEGF).^41^

#### 2.4.2 Second phase

There is currently a dearth of clinical data and experimental models characterizing the TIME of MRD. As mentioned above, the current studies conducted during the MRD phase have been completed in PDX models that lack a fully functional immune system. Hence, novel or improvised syngeneic mouse models which harbour the BRAF mutation are necessary to understand the contributing role of the TIME at the MRD phase. Currently, there are known to be two such syngeneic mouse models: Yale University Mouse Melanoma (YUMM) 1.1‐OVA‐Low (YOVAL1.1)^42^ and YUMM Exposed to Radiation (YUMMER1.7).^43^ Both models harbour the common mutations found in human cutaneous melanoma: *BRAF^V600E^*, *Pten^−/−^* and *Cdkn2a^−/−^* (Table 1). Accordingly, these models are found to respond to TT (BRAFi/MEKi) and ICI (α‐CTLA4 + α‐PD1)^42^, ^43^, ^44^ and, hence, are ideal models to understand the TIME changes and their interaction with the tumour cells at the MRD.

**TABLE 1:** Syngeneic BRAF mutant cutaneous melanoma mouse models

Full name	Abbreviated name	Mutation	Respond to TT and ICI	Ref
Yale University Mouse Melanoma 1.1 ‐OVA‐Low	YOVAL1.1	BRAF^V600E^, Pten^−/−^, Cdkn2a^−/−^	Yes	42
Yale University Mouse Melanoma 1.7 Exposed to Radiation	YUMMER1.7	BRAF^V600E^, Pten^−/−^, Cdkn2a^−/−^	Yes	43

Abbreviations: ICI, immune checkpoint inhibitors; TT, targeted therapy.

#### 2.4.3 Third phase

Pre‐clinical and clinical studies indicate that the resistance phase is defined by an immunosuppressive TIME characterized by increased infiltrates of immunosuppressive T‐reg cells^45^ and reduced melanoma antigen presentation and cytotoxic T‐cell infiltrates.^46^ Additionally, T‐cells upregulate their expression of immune exhaustion markers such as PD‐1 and TIM3, whereas melanoma cells and myeloid dendritic cells increase their expression of PD‐L1 and PD‐L2, respectively, thereby further suppressing the immune response.^45^, ^47^

Overall, these studies demonstrate that although initial treatment with TT induces an immunostimulatory TIME, the resistance phase is characterized by an immunosuppressive TIME. This highlights the importance of additional studies that characterize the TIME of MRD given the important role this phase may play in dictating the subsequent immunosuppressive TIME. Immunotherapeutic agents may also be used to increase the durability of TT by enhancing the initial immunostimulatory TIME and/or circumventing the immunosuppressive TIME.

## 3 IMMUNE CHECKPOINT INHIBITORS

Cutaneous melanoma is regarded as an immunogenic cancer typically with a high neoantigen burden. However, immunosuppressive mechanisms, including T‐reg cell infiltrates and inhibitory immune checkpoint molecules, limit the anti‐cancer immune response. Hence, targeting checkpoints through ICI, including α‐CTLA4 and α‐PD1, increases immune‐mediated clearance of melanoma cells.^48^, ^49^ Recent clinical trials with ICI have shown remarkable improvements in long‐term patient outcomes with a 6.5‐year OS rate of 52% for *BRAF*‐mutant melanoma; however, treatment was accompanied by high rates of toxicities, whereby grade 3 or 4 toxicities were observed in 53% of patients.^5^, ^50^, ^51^

**FIGURE 2:**
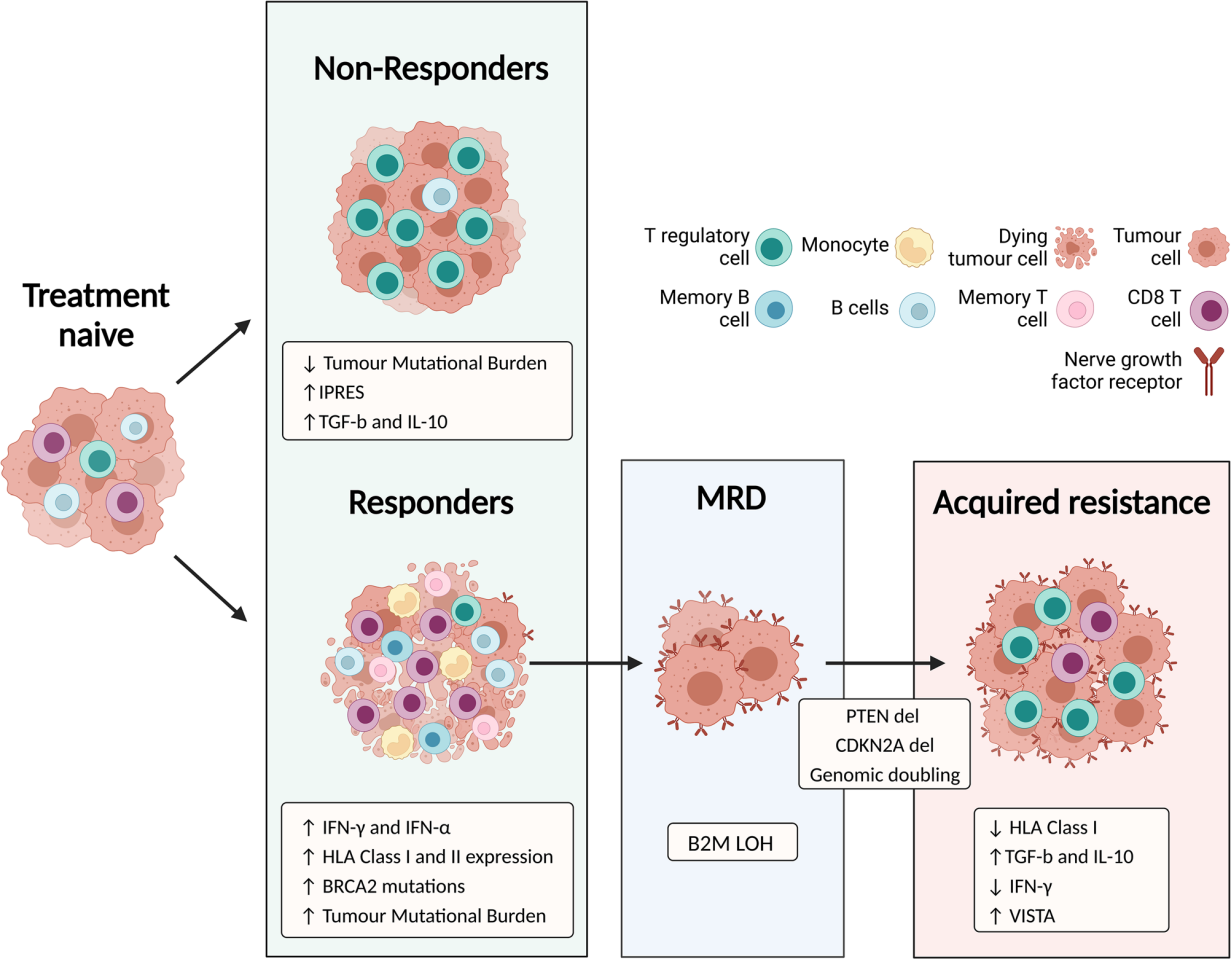
Response of cutaneous melanoma to immune checkpoint inhibition. Melanoma responds to immune checkpoint inhibitors (ICI) in three phases. In the first phase, tumours with sensitivity to ICI demonstrate early disease control, as shown by a reduction in tumour load. Biomarkers predictive of ICI sensitivity include an immunostimulatory tumour immune microenvironment (TIME) characterized by low infiltrates of T‐regulatory cells and high infiltrates of effector and memory B and T‐cells, and monocytes. Conversely, biomarkers predictive of primary ICI resistance include an immunosuppressive TIME defined by high levels of immunosuppressive T‐regulatory cells, TGF‐B and IL‐10 expression. In addition, although primary non‐responders are enriched for immunosuppressive signalling pathways such as the innate α‐PD1 resistance signature (IPRES), primary responders are enriched for immunostimulatory signalling pathways resulting in increased MHC, IFN‐g and IFN‐a expressions. Mutations in genes such as B2M also drive a high tumour mutational burden in primary responders, resulting in the generation of neoantigens which are targeted by the immune system. In the second phase, NGFR^hi^ tumour cells are recruited and persist whilst under therapeutic pressure. This is aided by multiple genomic changes, including beta‐2‐microglobulin (B2M) loss of heterozygosity (LOH), CDKN2A and phosphatase and tensin homolog (PTEN) deletion, which cover a survival and growth advantage. In the third phase, tumours with acquired resistance have been found to be enriched for NGFR^hi^ tumour cells, defects in IFN‐γ signalling and antigen presentation pathways, and an immunosuppressive TIME defined by high expression levels of TGF‐B and IL‐10, and high levels of T‐regulatory cells, the emergence and suppressive functions of which are induced by the V‐domain *I_g_* suppressor of T‐cell activation (VISTA) ligand.

Analogous to TT, the response of melanoma to ICI can also be conceptualized in three distinct phases (Figure 2). The *first phase* is characterized by early drug response whereby tumours with primary sensitivity to ICI show early disease control. Conversely, some tumours fail to respond to ICI due to primary resistance. It can be postulated that the *second phase* is defined by therapeutic tolerance, whereby residual tumour cells persist whilst under therapeutic pressure, establishing MRD. This is akin to the ‘equilibrium’ phase proposed by Schreiber, a latent period in which a balance is struck between tumour clearance and tumour growth.^52^ The *third phase* is characterized by disease progression secondary to the development of acquired resistance to ICI.

### 3.1 First phase: responders versus non‐responders

During the first phase, patients can be divided into ‘primary responders’ or ‘primary non‐responders’ depending on their sensitivity to ICI (Figure 2). This is typically assessed via the widely used conventional Response Evaluation Criteria in Solid Tumours (RECIST)^53^ or immune‐related response assessment criteria (iRECIST),^54^ which evaluates tumour responsiveness to therapeutics based on criteria such as tumour diameter and lymph node involvement. Accordingly, some biomarkers have been identified that distinguish primary responders from primary non‐responders.

Multiple studies have demonstrated a positive correlation between ICI responsiveness and OS with tumour mutational burden (TMB) and neoantigen load.^55^, ^56^, ^57^ One study showed that in responding patients, an expansion of T‐cell clones was accompanied by a reduction in TMB and neoantigen load, suggesting that tumours with a high number of neoantigens are better able to elicit immune‐driven recognition and elimination of tumour cells with a high number of neoantigens.^57^ Additionally, a subset of primary responders harboured mutations in *BRCA2* that further increased the neoantigen load.^56^, ^58^ Responders also heighten the expression of immunostimulatory genes, including *MHC I/II* ^59,60^ and *IFN‐γ/α*, resulting in increased cytotoxic activity.^60^, ^61^ In contrast, primary resistant tumours upregulate immunosuppressive signalling pathways, including sphingosine kinase‐1 (SK1) resulting in a reduced CD8/T‐reg cell ratio and increased immunosuppressive cytokine expression,^62^ WNT/β‐catenin resulting in dendritic cell and T‐cell exclusion^63^ and IFN‐γ signalling pathway defects resulting in attenuated tumour cell apoptosis and growth suppression.^64^ Non‐responsive patients also harbour the unique innate anti‐PD1 resistance signature (IPRES) comprising a group of 26 transcriptional signatures, which drive resistance through processes such as angiogenesis and epithelial‐to‐mesenchymal transition.^56^

Furthermore, differences in the composition of TIME have been identified in primary responders compared to non‐responders. Responsive tumours have an immunostimulatory TIME characterized by high levels of monocytes^65^ and tumour infiltrating lymphocytes (TILs), including memory B‐cells,^66^ effector memory T‐cells^67^ and activated cytotoxic T‐cells.^68^ Conversely, tumours with intrinsic resistance are characterized by an immunosuppressive TIME marked by low levels of TILs, B‐cells, natural killer cells^69^ and CD8 T‐cells with increased levels of T‐reg cells and immunosuppressive cytokines, including TGF‐B and IL‐10.^62^

This suggests that certain biomarkers, including a high TMB, an immunostimulatory TIME and an activation of immunostimulatory signalling pathways, predict primary sensitivity to ICI. Conversely, patients with primary resistance to ICI have an immunosuppressive TIME and upregulate immunosuppressive signalling pathways.

### 3.2 Second phase: minimal residual disease

Based on the frequency of patients who progress after partial or complete response to ICI, we propose that a portion of patients who initially respond to ICI go on to establish MRD.^70^ These cells persist under therapeutic pressure eventually driving disease progression and secondary resistance. Unfortunately, there is a scarcity of studies that have been conducted characterizing the biology during MRD associated with ICI.

One study that conducted serial biopsies of a patient during ICI demonstrated, through whole exome sequencing and RNA sequencing, the presence of seven dominant tumour clones on initiation of treatment. However, during the MRD phase, an *NGFR^hi^* clone characterized by 15q deletion (including beta 2 macroglobulin [*B2M*]) acquired additional genetic alternations, including the deletion of *CDKN2A* and phosphatase and tensin homolog (*PTEN*) genes.^71^ These changes likely increased the therapeutic resistance of these cells, as the *NGFR^hi^* clone was found to dominate tumour biopsies during the third progression phase. The authors proposed that the immune system may have played a role in pruning susceptible clones given that cells of the dominant lineage acquired immune‐evasive adaptations.^72^

Interestingly, this study, similar to studies with TT, suggested that during treatment with ICI, a genetic clone is selected, which undergoes additional transcriptional changes driving therapeutic resistance and disease progression. Additional studies, however, are required to characterize the mechanisms at play during MRD after treatment with ICI to better understand how this phase may be targeted therapeutically.

### 3.3 Third phase: acquired resistance

Following MRD or partial response, some patients acquire resistance to ICI, with 12%–38% of those who initially respond eventually progressing on therapy.^73^ Several mechanisms of acquired resistance have been identified that ultimately drive disease progression, including mutations and/or loss of heterozygosity (LOH) in *B2M*, a key component of MHC Class I, which results in loss of antigen presentation; LOH of *JAK1* and *JAK2* proteins resulting in loss of sensitivity to IFN‐mediated growth inhibition and immune‐mediated death^74^, ^75^; and loss of the tumour suppressor gene *PTEN* associated with decreased T‐cell infiltration and increased expression of immunosuppressive cytokines.^76^, ^77^ The expression of co‐inhibitory receptors on immune cells in the TIME such as VISTA is another mechanism of acquired resistance. Biopsies taken at the time of acquired resistance revealed a 67% increase in the expression of VISTA on lymphocytes compared to pre‐treatment, which was accompanied by a significant increase in intratumoral FOXP3+ T‐reg lymphocyte infiltrates.^78^ Furthermore, as aforementioned, a recent study revealed that a tumour cellular sub‐type characterized by an NGFR^hi^PDL1^hi^ signature is responsible for driving therapeutic resistance and disease progression. During phase 3, an immunosuppressive TIME has been found to surround these cells characterized by a high influx of T‐reg cells.^72^

Given these diverse mechanisms of acquired resistance, no uniform biomarkers predictive of therapeutic sensitivities have been identified to date. To achieve this, longitudinal studies characterizing MRD and acquired resistance in patient cohorts are needed. This requires the multicentre coordination of recruitment and methodologies to ensure adequate patient numbers.

## 4 RATIONAL SEQUENCING OF TARGETED THERAPY, IMMUNOTHERAPY AND CROSS‐RESISTANCE

Both TT and ICI have their own therapeutic advantages and disadvantages. Although TTs generate outstanding initial ORR, their effects are usually relatively short‐lived due to acquired resistance. Conversely, although ICIs have lower initial ORR, they induce more durable responses. Given these complementary effects, sequential treatment has been proposed to maximize clinical outcomes whilst circumventing potential toxicities associated with combinatorial treatment. The question remains, however, of which therapy to initiate first. As such, prospective clinical trials have sought to compare therapeutic responses of ICI and TT as first‐ or second‐line treatments in BRAF‐mutant melanoma.

### 4.1 Switching therapy upon progression

**FIGURE 3:**
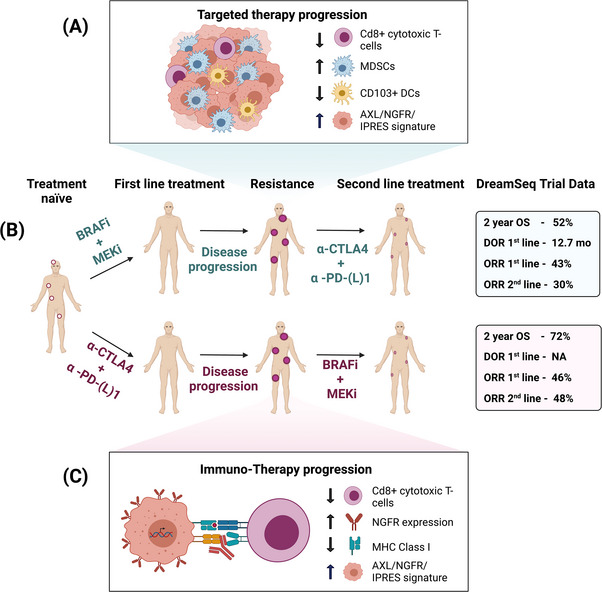
Cross‐resistance between immunotherapy and targeted therapy (TT): (A) tumours that progress on TT are enriched for an immunosuppressive tumour immune microenvironment (TIME) characterized by high infiltrates of myeloid‐derived suppressor cells (MDSCS) and low infiltrates of CD8+ T‐cells and CD103+ DCs, all of which hamper therapeutic responses to subsequent immunotherapy; (B) preliminary results from the DREAMseq trial indicate improved 2‐year overall survival (OS) and progression‐free survival (PFS) rates with first‐line α‐CTLA4 + α‐PD1 followed by BRAFi + MEKi compared with first‐line BRAFi + MEKi followed by α‐CTLA4 + α‐PD1; (C) tumours that progress on immune checkpoint inhibitors (ICI) are found to be enriched for NGFR^hi^ cells and an Innate Α‐PD1 Resistance (IPRES) Signature but express low levels of MHC and CD8+ T‐cells, all of which hamper therapeutic responses to second‐line TT.

Several prospective clinical trials are investigating various treatment sequences with the view to determining optimal front‐line treatment. The DREAMseq clinical trial (NCT02224781) has randomly assigned patients to two treatment sequences: Sequence 1 comprises patients commenced on ICI (Arm A) followed by a switch to TT (Arm C) on progression; sequence 2 comprises patients commenced on TT (Arm B) followed by a switch to ICI (Arm D) on progression (Figure 3B). Although upfront ICI (Arm A) and TT (Arm B) had comparable ORRs of 46% and 43%, respectively, the effectiveness of second‐line ICI (Arm D) appeared dampened with an ORR of 30% compared to second‐line TT (Arm C) with an ORR of 48%. Additionally, interim results trended towards improved median and landmark PFS at a median follow‐up of 27.7 months in those commenced on first‐line ICI (*p* = .054), with a 20% improvement in 2‐year OS (*p* = .0095)^79^ (Table 2).

**TABLE 2:** Key characteristics of prospective clinical trials investigating treatment sequence strategies

Study	Experimental arm	PFS (%)	ORR (%)	OS (%)	Ref
DREAMseq	Arms A and C (ICB → AR → TT)	First line –41.9	First line – 46 Second line – 47.8	66.2	79
	Arms B and D (TT → AR → ICI)	First line – 19.2	First line – 43 Second line – 29.6	42.8	
SECOMBIT	Arm A (TT → AR → ICI)	41	First line – 87 Second line – 25.7	54	80
	Arm B (ICI → AR → TT)	53	First line – 44.9 Second line – 57.9	62	
	Arm C (TT → ICI → AR → TT)	54	First line – 82.4 Second line – 62.2	60	

Abbreviations: AR, acquired resistance; ICI, Immune Checkpoint Inhibitors (α‐CTLA4 + α‐PD1); ORR, overall response rate; OS, overall survival rate; PFS, progression free survival; TT, targeted therapies (BRAFi + MEKi).

In another clinical trial (SECOMBIT: NCT02631447), patients will be randomized to one of three treatment arms: Arm A (TT first line followed by ICI upon progression); Arm B (ICI first line followed by TT upon progression); Arm C (8‐week run‐in of TT, followed by ICI until progression, followed by a switch to TT). Preliminary results from SECOMBIT show an ORR for first‐line treatment of 87% and 47% in Arms A and B, respectively. Upon progression, the ORR for second‐line treatment was 25% and 61%, respectively. This suggests an inferior ORR for ICI when given after TT compared to ICI in therapy naïve patients. The 3‐year ‘total’ PFS, or time to second progression, was 41% and 53% in Arms A and B, respectively, and the OS at 3 years was 54% and 62% in Arms A and B, respectively. Arm C, an approach that reflects switching therapy with MRD gave a 3‐year PFS and OS of 54% and 60%, respectively^80^ (Table 2).

Hence, akin to DREAMseq, data from SECOMBIT suggested improved clinical outcomes with first‐line ICI. These clinical trials also suggest that melanoma progression on first‐line TT may promote relative resistance to second‐line ICI, referred to as ‘cross‐resistance’. However, the biology of cross‐resistance remains to be adequately characterized.

### 4.2 Cross‐resistance

A recent study utilizing a mouse model showed that tumours with acquired resistance to TT are also subsequently resistant to ICI due to an overall immunosuppressive TIME (Figure 3A). These cross‐resistant tumours have reduced CD8+ T‐cell infiltrates, downregulated an expression of T‐cell effector molecules and markers of activation, impaired maturation and functionality of CD103+ dendritic cells and increased immunosuppressive myeloid cells, all of which are supported by observations from other clinical data sets.^45^, ^81^, ^82^ The authors found that the overactivation of the MAPK pathway underpinned cross‐resistance through the establishment of an immunosuppressive TIME. Conversely, another study demonstrated that cross‐resistance is independent of MAPK but rather hinges on the upregulation of RTKs which drive an immune‐suppressive, mesenchymal and angiogenic state.^47^ Clinical data from SECOMBIT and DREAMseq trials also support this concept of cross‐resistance. Both studies demonstrated that ICI appears to be less effective when used after disease progression on TT^79^, ^80^.

Although not investigated experimentally, multiple gene expression signatures are shared between melanomas resistant to TT with those resistant to ICI, such as IPRES.^83^ This suggests that tumours with innate resistance to ICI may also be resistant to TT, suggesting another possible mechanism of cross‐resistance. Similarly, two other studies have demonstrated that the NGFR^hi^ phenotype predicts resistance to TT and ICI, highlighting yet another mechanism of cross‐resistance ^72^, ^84^ (Figure 3C).

Therefore, although sequencing may assist in maximizing the therapeutic potential of TT and ICI, the emergence of cross‐resistance remains an issue. Additional biological studies are, thus, needed to investigate the mechanisms of cross‐resistance and how they may be overcome to re‐sensitize patients to sequential therapy.

## 5 INTERVENING AT THE MINIMAL RESIDUAL DISEASE

As discussed in this paper, we propose that the MRD phase plays a critical role in determining the ultimate therapeutic response to TT and ICI, by providing a nidus for the development of acquired resistance. Additionally, melanoma may be at its most vulnerable during MRD due to a low tumour load. Therefore, additional therapeutic interventions during MRD may enable long‐term disease control, thereby ultimately achieving a progression‐free state or clinical cure. In doing so, these therapeutics would help reduce the risk of disease reoccurrence under TT and ICI pressure, which differs markedly from current adjuvant therapies, which seek to combat reoccurrence secondary to surgical resection under immune‐system pressure.

Data from Arm C of the SECOMBIT trial, in which patients were switched to ICI during the MRD phase of TT, showed similar OS and total PFS to Arm B (ICI until progression followed by TT), which was superior to Arm A (TT until progression followed by ICI). This highlights that both switching during the MRD phase or ICI followed by TT may be effective therapeutic approaches for melanoma. Results from SECOMBIT's ongoing biomarker analysis will help provide an understanding of the biological mechanisms at play during the response, MRD and resistance phases, by defining genetic, non‐genetic and changes in the TIME. Particular attention should be directed towards the emergence of the *NCSC* phenotype, which, as outlined, is responsible for non‐genetic mechanisms of acquired resistance and immune evasion.

## 6 CONCLUSIONS AND FUTURE DIRECTIONS

Over the last decade, the introduction of BRAF/MEK‐TT and ICI has revolutionized the treatment landscape of cutaneous melanoma, dramatically improving patient outcomes. Although TTs have an outstanding ORR, ICIs have durable PFS owing to a robust clinical response. Unfortunately, however, both intrinsic and acquired resistances remain a significant concern for ICI. Both upfront combinatorial treatment and sequencing upon progression have been explored as possible treatment approaches for maximizing the therapeutic impact of ICI and TT. Although retrospective studies and clinical trials show the benefit of triplet therapy (BRAFi + MEKi + ICI) over single‐modality therapies, and upfront ICI followed by TT upon progression, additional mechanistic studies are required to study the biology underpinning these approaches to maximize the benefit.

Both TT and ICI have an MRD phase from which disease relapse emerges through a host of genetic and non‐genetic alterations. We suggest that further studies are needed to adequately characterize the origin and evolution of resistance during the MRD phase, for example the emergence of NCSCs, with the view to developing potentially curative treatment options. The clonal evolution of melanoma following therapeutic intervention could be assessed through single cell‐RNA sequencing or clonal mapping of serial tumour biopsies. Importantly, given that the emergence and expansion of NCSCs have been shown to be independent of a tumour's initial driver mutations, these studies may also have implications for BRAF‐non‐mutant melanoma.^16^ In addition, the TIME of the MRD may be characterized to ascertain uniform biomarkers that can be targeted therapeutically. The emergence of molecular spatial profiling supported by existing single‐cell technologies such as single‐cell RNA transcriptomics or multi‐parameter flow/mass cytometry may also offer significant insights into relationships between cells. Through the use of these technologies, novel therapeutics may be developed which prevent the emergence of resistance and disease progression.

## CONFLICT OF INTEREST STATEMENT

There are no conflicts of interest to disclose.

## HUMAN AND ANIMAL RIGHTS AND INFORMED CONSENT

This article does not contain any studies with human or animal subjects performed by any of the authors.
